# De-adoption and its 43 related terms: harmonizing low-value care terminology

**DOI:** 10.1186/s12916-015-0511-4

**Published:** 2015-10-20

**Authors:** Danijela Gnjidic, Adam G. Elshaug

**Affiliations:** Faculty of Pharmacy, University of Sydney, Sydney, NSW Australia; Menzies Centre for Health Policy, University of Sydney, Sydney, NSW Australia; Lown Institute, Brookline, MA USA

**Keywords:** De-adoption, Harmful, Ineffective, Low-value care, Medical interventions

## Abstract

Research into the prevalence and impact of low-value medical practices has evolved substantially over the past two decades. However, despite international efforts, many challenges still remain with regards to progress in this field, including limits in the capacity to identify and prioritize low-value care practices and to systematically appraise clinical and policy attempts at redressing low-value care. A recent article by Niven et al. in *BMC Medicine* consolidates the current literature and terminology on the de-adoption of clinical practices, advocating the use of de-adoption as an appropriate term to label low-value care and proposes a new synthesis model to facilitate efforts to reverse ineffective and harmful medical practices. We hope that this work will facilitate advances in low-value care research and policy, and shift focus towards establishing evidence for de-adopting low-value interventions, which is crucial since attempts to reduce low-value care interventions have shown mixed results.

Please see related article: http://www.biomedcentral.com/1741-7015/13/255

## Background

**Fig. 1:**
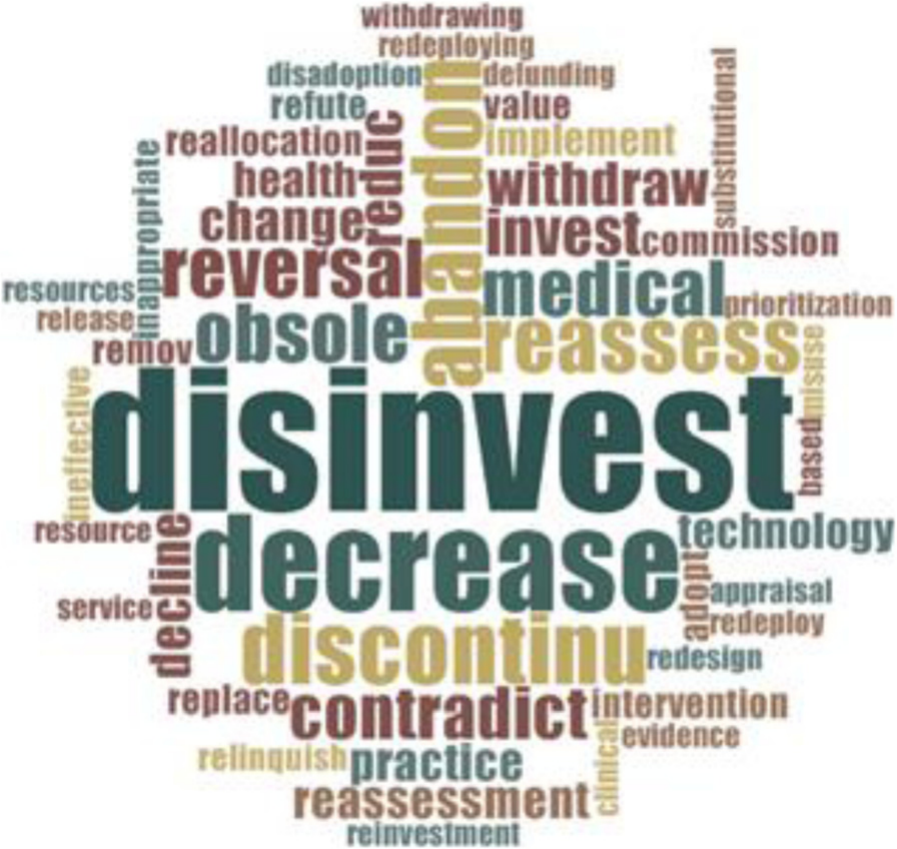
Word cloud of frequency of terms used to label low-value care practices and policy processes, derived by entering the 43 terms identified by Nieven et al. [3] in nVivo software

In the 1990s, England’s National Institute for Health and Care Excellence co-opted the term ‘disinvestment’ from industry parlance, heralding its transition to the health sector [1]. Within industrial settings, disinvestment primarily refers to the removal of resources from obsolete items such as machinery. In healthcare settings, there is less scope for such binary verdicts and, as such, more attention is given to the complex issues associated with the ethics of waste reduction. The most common definition places it as “*processes of withdrawing (partially or completely) health resources from any existing health care practices, procedures, technologies or pharmaceuticals that are deemed to deliver little or no health gain for their cost, and are thus not efficient health resource allocations*” [2], allowing for resource re-allocation. As Niven et al. [3] so aptly indicate, many related concepts are subsumed within misuse, overtreatment, overdiagnosis, overmedicalization, waste, opportunity cost, allocative and/or technical efficiency, resource re-allocation, and de-adoption. Terminological proliferation has ensued for several years, as visualized in the word cloud presented herein (Fig. 1) and derived from the most common terms identified by Niven et al. [3].

The article by Niven et al. [3] represents an important contribution to the field, representing, along with the manuscripts referenced within it, a valuable repository cataloguing the current state-of-the-science from around the world with regards to efforts at reducing the use of low-value healthcare. Throughout article we are reminded that contemporary de-adoption initiatives can be likened to ‘old wine in a new bottle’, for related programs have emerged and re-emerged since the 1970s [4, 5]. While the desire to minimize waste and deliver safe, effective, and efficient healthcare is old wine, the new bottle is represented by ever-evolving research, analysis, health technology assessment methods, and dovetailed policy processes. It is clear that the clinical, research, and policy communities have attended to the successes and failures of the past and are evolving to develop more robust methods moving forward. Further, many of the challenges faced are universal (e.g. sources of resistance to a potential loss function, burden of evidence requirements, levers to encourage optimal use), yet initiatives tend to be context specific: there is no one-size-fits-all approach and, for numerous evolving programs, their success remains uncertain.

Nevertheless, despite increasing international efforts, researchers are still faced with significant challenges with regards to research on low-value clinical practices [6]. Curiously, the ability to identify and prioritize which practices are of low-value appears to have overtaken the ability to systematically evaluate the clinical and policy attempts at reducing low-value care [7]. Indeed, strategies such as Choosing Wisely have been established in the USA, UK, Canada and, recently, Australia to identify and (it is to be assumed) reduce the overuse of interventions considered as low-value. To illustrate some examples of low-value interventions we present the International Choosing Wisely Top 10 list [8].

- Imaging for low back pain
- Stress cardiac imaging for initial evaluation
- Annual stress cardiac imaging
- Pre-op testing before low risk surgery
- Antibiotics for sinusitis
- Benzodiazepines in the elderly
- Long-term proton-pump inhibitor therapy for gastrointestinal symptoms
- Antipsychotics for dementia
- Antimicrobials for bacteriuria in elderly
- Urinary catheters

For instance, overuse of pharmaceutical agents of questionable benefit, such as antipsychotics among people with dementia, has been of particular concern. Despite strong evidence that antipsychotic use is linked with significant harms and that the cessation or deprescribing of antipsychotics is safe, these agents continue to be overused among people with dementia [9]. Efforts are needed to establish evidence-based guidelines for the most effective interventions to deprescribe antipsychotics and other medications of questionable benefit in this patient group [10, 11].

Given this background, Niven et al.’s scoping review [3] consolidates the current literature and terminology on the de-adoption of clinical practices, with the ultimate aim to develop a new synthesis model for providers and decision-makers to facilitate the reversal of ineffective and harmful medical practices. The authors identified 43 different terms used to refer to the process of de-adoption, with ‘disinvest’ (39 %) and ‘decrease use’ (24 %) being the most frequently cited terms. They further recommend that ‘de-adoption’ should be the term used to standardize the literature on low-value clinical care. Given the increasing focus on generating evidence to inform low-value care practices, we agree that reaching a consensus on the most appropriate term to be used to refer to low-value care is of benefit. We recommend that researchers debate the use of the ‘de-adoption’ term to standardize the terminology [6]. Interestingly, de-adopt was only cited by 3 % of the included papers. It remains to be seen whether this will impose some barriers to implementing the ‘de-adoption’ term in the relevant literature.

Niven et al. [3] also indicate that the most prominent low-value care ‘red flag’ in their sourced studies was that of safety concerns. While legitimate, the onus is on clinicians and researchers to conduct further studies to generate evidence about low-value practices based on their ineffectiveness rather than the red flag of safety alone. To further progress the field, the authors propose a de-adoption framework model based on 13 frameworks that conceptualize individual components of a de-adoption process. The question is, how can attention be focused towards the implementation of this framework to guide the de-adoption of ineffective and harmful practices? Each framework component must be considered to understand the issues that underpin this model and be able to answer the question above.

## Untangling the de-adoption framework

Historically, the primary focus lay on identifying low-value clinical practices; focus has now shifted to prioritizing selected low-value practices for evidence-based review or reassessment [7, 12]. The assessment or measurement of the prevalence of low-value practices has recently received significant attention. While these methods are evolving, there remains a need for considerable further measurement worldwide [6]. Additionally, the design and implementation of de-adoption initiatives at the clinical and policy levels is no simple task. Arguably, there is no one model to drive this step given that the circumstances of individual healthcare environments are widely varied and context specific [13]. For example, as the recent OECD report reveals [14], individual countries have tackled the problem of cesarean delivery over-use differently, all with varying degrees of success. Niven et al. [3] further emphasize this point in their synthesis. So too, rather than re-inventing the wheel, the ‘adaptation of a knowledge step’ in the Niven model is well founded, with growing examples of existing policy processes that can be merged with promising initiatives, thus adding value to the overall process. The next step in their model involves the evaluation of de-adoption processes and outcomes [15]. Without doubt, there is a dearth of information in this domain due to the lack of a publication imperative by policy stakeholders performing this work and/or due to evidence lying in grey literature that is difficult to obtain. Sustaining de-adoption initiatives is particularly challenging and it is well regarded in the field that de-adoption is far from merely reversing the implementation process [6]. The last step of this framework, namely the assessment of barriers and facilitators to the de-adoption of interventions, is perhaps the most important. While this framework step has received a great deal of attention from both the clinical and policy analysis perspectives [2, 16, 17], it remains under-represented in terms on quantitative evaluation.

## Conclusions

We commend the authors for summarizing the current literature on low-value clinical practices, the terminology used thus far, and the impact of de-adoption interventions. This scoping review substantially contributes to the continuing maturation of low-value clinical practice literature. This is an important step in consolidating the research to date, particularly regarding what constitutes low-value care, and is essential to generate the evidence base for de-adoption approaches of clinical practice.
